# Chronic osteomyelitis post fracture

**DOI:** 10.11604/pamj.2021.39.76.29612

**Published:** 2021-05-26

**Authors:** Om Chandrakant Wadhokar, Waqar Mohsin Naqvi

**Affiliations:** 1 Department of Musculoskeletal Physiotherapy, Ravi Nair Physiotherapy College, Datta Meghe Institute of Medical Sciences, Sawangi(M), Wardha, India,; 2 Department of Community Health Physiotherapy, Ravi Nair Physiotherapy College, Datta Meghe Institute of Medical Sciences, Sawangi(M), Wardha, India

**Keywords:** Chronic osteomyelitis, diabetes mellitus, adult osteomyelitis

## Image in medicine

A 45-years-old male met with a road traffic accident for which he got admitted to Acharya Vinoba Bhave Rural Hospital (AVBRH). On admission X-ray was done; which revealed multiple fracture of the right femur. This patient was operated by open reduction and internal fixation, the individual is a known diabetic since 5 years and is on regular medication for the same. The limb was then immobilized in the cast and the patient was discharged after one month, when he came back for plaster removal. The patient complains of pain in the right thigh, fever, weakness and enlarged femoral lymph nodes. Radiography was done, which revealed formation of involucrum (A,B). Later white blood cell (WBC) count and X-ray was done to confirm the diagnosis. The individual mention the history of working in the farm. After 3 days as mentioned by patient, he had fever. He consulted a general physician who gave him medication.

**Figure 1:**
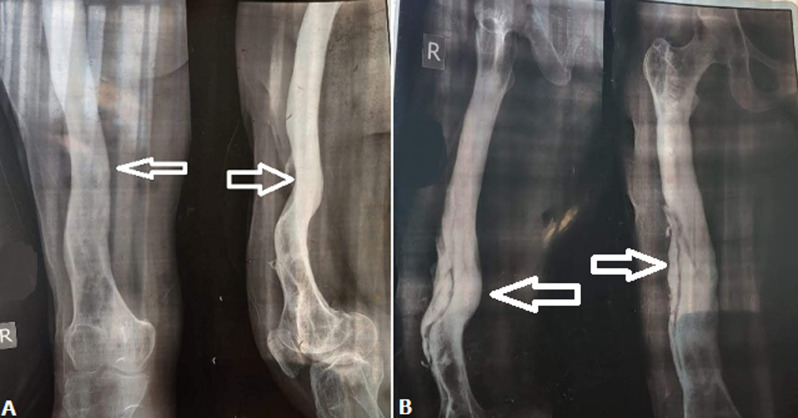
showing the radiological findings of right femur; A) showing the antero posterior view of right femur; B) showing the lateral view of the right femur

